# Did Environmental and Climatic Factors Influence the Outcome of the COVID-19 Pandemic in the Republic of Serbia?

**DOI:** 10.3390/healthcare13131589

**Published:** 2025-07-02

**Authors:** Milos Gostimirovic, Ljiljana Gojkovic Bukarica, Jovana Rajkovic, Igor Zivkovic, Ana Bukarica, Dusko Terzic

**Affiliations:** 1 Institute for Pharmacology, Clinical Pharmacology and Toxicology, Faculty of Medicine, University of Belgrade, 11000 Belgrade, Serbia; milos.gostimirovic@med.bg.ac.rs (M.G.); bukarica@rcub.bg.ac.rs (L.G.B.); 2 Institute for Cardiovascular Diseases Dedinje, Faculty of Medicine, University of Belgrade, 11000 Belgrade, Serbia; igor88zivkovic@gmail.com (I.Z.); ana.bukarica@institutdedinje.org (A.B.); 3 Clinic for Cardiosurgery, Clinical Centre of Serbia, 11000 Belgrade, Serbia; terzic.dusko@gmail.com

**Keywords:** air pollution, climate factors, COVID-19 pandemic, health effects, Republic of Serbia

## Abstract

**Background**: The aim of the study is to determine whether environmental and climatic factors (air quality, precipitation rates, and air temperatures) alongside specific public health measures (social distancing and vaccination) have influenced total number of SARS CoV-2 positive cases (TOTAL CASES) and deaths (TOTAL DEATHS) from COVID-19 infection in the Republic of Serbia (RS). **Method**: An observational, retrospective study was conducted, covering the following three-year period in the RS: I (1 March 2020–1 March 2021); II (1 March 2021–1 March 2022); and III (1 March 2022–1 March 2023). Air quality was expressed as the values of the air quality index (AQI) and the concentrations of particulate matter 2.5 µm (PM_2.5_). Precipitation rates (PREC) were expressed as the average monthly amount of rainfall (mm), while average air temperatures (AIR TEMP) were expressed in °C. Data were collected from relevant official and publicly available national and international resources. Data regarding the COVID-19 pandemic were collected from the World Health Organization. **Results**: No differences between the periods were observed for the average values of AIR TEMP (11.2–12.2 °C), PREC (56.1–66.8 mm), and AQI (57.2–58.8), while the average values of PM_2.5_ significantly decreased in the III period (21.2 compared to 25.2, *p* = 0.03). Both TOTAL CASES and TOTAL DEATHS from COVID-19 infection showed positive correlation with the AQI and PM_2.5_ and a negative correlation with the AIR TEMP. The correlation coefficient was strongest between TOTAL DEATHS and the AIR TEMP in the II period (r = −0.7; *p* = 0.007). The extent of rainfall and vaccination rates did not affect any of the observed variables. No differences in TOTAL CASES and TOTAL DEATHS were observed between the periods of increased social measures and other months, while both statistically significantly increased during the vaccination period compared to months without the vaccination campaign (*p* < 0.02, for both). **Conclusions**: Air quality, more precisely AQI and PM_2.5_ and average air temperatures, but no precipitation rates, influenced the number of TOTAL CASES and TOTAL DEATHS from COVID-19 infection. These were the highest during the vaccination period, but vaccination could be considered as a confounding factor since the intensive vaccination campaign was conducted during the most severe phase of the COVID-19 pandemic. Social distancing measures did not reduce the number of TOTAL CASES or TOTAL DEATHS during the COVID-19 pandemic.

## 1. Introduction

The COVID-19 pandemic, caused by the SARS-CoV-2 virus, has been one of the largest global health crises in modern history [1]. Five years after the official start of the pandemic, the health consequences of the so-called post-COVID-19 syndrome are still reported in the literature around the world. This disease starts several weeks or months after acute infection, presenting with chronic fatigue, shortness of breath, sleep disorders, rapid heartbeat, and depression, probably as a consequence of inflammation, hormonal imbalance, or microscopic thrombosis. In a broader context, this all has an impact on the national economy, which is why the problem of COVID-19 infection is still ongoing [2,3].

The Republic of Serbia (RS) had a case fatality rate (CFR) (deaths/infected ratio) from COVID-19 infection of 0.69% and was ranked among the countries with a low CFR. However, if we consider the total mortality rate (deaths/total population ratio), with a rate of 2735 deaths per million inhabitants, the RS moves into the top half of the world list (45th place out of 231 countries), which is a worryingly high position [4].

Such a relatively high mortality rate is quite unusual, especially considering the fact that the country was highly equipped in terms of diagnostic capabilities, infrastructural capacities, and modern therapeutic care for COVID-19 patients, expressed as a sufficient number of ventilators, modern laboratories for the detection of the SARS-CoV-2 virus, RT-PCR tests, innovative therapy, and wholehearted application of the world’s treatment protocols by the many engaged physicians and experts [5].

Considering the nature of infectious diseases, the question arises whether some external factors have influenced the degree of transmission and mortality from COVID-19 infection, especially since the RS is a country with numerous unresolved environmental and climatic issues. For example, the RS is a country with unfavorable air quality, extremes in terms of average air temperatures, increasingly mild winters, long-lasting droughts, and heat waves, with a minimal amount of precipitation, even during the spring [6,7]. It seems that air quality and average values of air temperature and precipitation rates stand out as the most important factors, and the harmful health effects of these have been demonstrated in numerous national studies [8,9,10].

Numerous studies around the world report a clear correlation between the abovementioned environmental and climatic factors and society’s burden from COVID-19 infection, but the direction and intensity of this correlation are different in different parts of the world. Most of them report an inverse (negative) correlation with average air temperatures and precipitation rates and a direct (positive) correlation with air quality [11,12,13,14,15].

In order to mitigate the negative impact on people’s health, the course of the pandemic was also marked by periods of restrictive social measures regarding limitation of physical contact, and the beginning of 2021 was also marked by intensive vaccinations around the world. Both of these factors could have influenced the final outcome of COVID-19 infection [16,17,18].

The aim of this paper is to determine the influence of environmental and climatic factors on the total number of cases and total number of deaths from COVID-19 infection in the RS, evaluating air quality (AQ), average air temperatures (AIR TEMP), and precipitation rates (PREC). Additionally, we explored the impact of specific public health measures (the period of increased social distancing and the vaccination period) on the observed variables.

## 2. Material and Methods

### 2.1. Research Design

This research is observational and retrospective, covering a three-year period (36 months) in the RS, from 1 March 2020 to 1 March 2023. In order to facilitate the correlation of all relevant data, the entire period has been divided into three sub-periods:

- Period I (1 March 2020–1 March 2021);
- Period II (1 March 2021–1 March 2022);
- Period III (1 March 2022–1 March 2023).

### 2.2. Data Collection

Publicly available sources were used, which included official databases on daily confirmed SARS-CoV-2-positive cases and deaths and environmental and meteorological data (AIR TEMP, PREC, and AQ).

Independent variables included the following:

- Environmental factors: Air pollution, objectified as air quality index (AQI) and concentrations of particulate matter 2.5 µm (PM_2.5_). Specifically, the concentrations of other pollutants: particulate matter 10 µm (PM_10_), sulfur dioxide (SO_2_), nitrogen dioxide (NO_2_) (in µg/m^3^), and heavy metals (lead (Pb), arsenic (As), cadmium (Cd), and nickel (Ni)) (in ng/m^3^).
- Climatic factors: AIR TEMP and PREC are objectified as average air temperatures (in °C) and average precipitation rates (in mm).

Dependent variables included the total number of confirmed SARS-CoV-2-positive cases and total number of deaths from COVID-19 infection (expressed as absolute numbers).

All data were presented on a monthly level. Official data on environmental and climate factors in the included reports were presented from the 1st to 31st of a month. Therefore, our study began six days before the COVID-19 pandemic officially started in the RS (1 March 2020, instead of 6 March 2020).

As mentioned, data sources were collected from official and publicly available international and national repositories and may be divided into the following specific categories:

1. Climatic factors (AIR TEMP and PREC)—Annual reports from the Republic Hydro-meteorological Service of the RS, for 2020–2023 [19,20,21,22].

Data of AIR TEMP and PREC were collected from 28 locations of RS, geographically divided as follows: north—Palić, Sombor, Novi Sad, Zrenjanin, Kikinda, and Banatski Karlovac; west—Loznica, Sremska Mitrovica, and Valjevo; central—Beograd, Kragujevac, Kraljevo, Ćuprija, and Sremska Palanka; east—Veliko Gradište, Crni vrh, Zaječar, Dimitrovgrad, and Negotin; south—Zlatibor, Sjenica, Požega, Kopaonik, Kuršumlija, Kruševac, Niš, Leskovac, and Vranje. It included both plains and mountainous areas.

2. Environmental factor (air pollution)—AQI and concentrations of PM_2.5_—World Air Quality Reports (IQAir) [23]; concentrations of PM_10_, SO_2_, NO_2_, Pb, As, Cd, and Ni—Annual Report on the Air Quality in the Republic of Serbia, Serbian Environmental Protection Agency (SEPA), Ministry of Environmental Protection, Republic of Serbia [24,25,26,27].

Due to its exceptional importance for the development of diseases, PM_2.5_ concentrations are shown separately from the other particles (see Section 3).

Concentrations of PM_10_, SO_2_, NO_2_, Pb, As, Cd, and Ni were presented as the average annual values for the three consecutive years—2020, 2021, 2022, and 2023. Therefore, subdivision into three periods was not applied here.

Good AQ was represented by an AQI < 50 (or PM_2.5_ < 25), whereas 51 < AQI < 100 (or 25 < PM_2.5_ < 50) represented a moderately unhealthy AQ. Upper annual allowed concentrations for PM_10_, SO_2_, NO_2_, Pb, As, Cd, and Ni in the air were as follows: 50 µg/m^3^, 50 µg/m^3^, 40 µg/m^3^, 0.5 µg/m^3^, 6 ng/m^3^, 5 ng/m^3^, and 20 ng/m^3^.

Average annual daily concentrations of these pollutants in the RS are calculated according to aforementioned reports of the SEPA and represent average annual concentrations in several measuring stations in the RS (15–68, see Table 3), divided by the years. The mean annual concentration of the corresponding pollutant is calculated as follows: sum of the measured concentrations in the individual measuring station divided by the number of measuring stations in the corresponding year.

3. Burden of the COVID-19 pandemic (total number of confirmed SARS-CoV-2-positive cases and total deaths from COVID-19 infection) in the RS for 2020–2023—World Health Organization (WHO) [28], Ministry of Health of Republic of Serbia [29], Institute of Public Health of Serbia “Dr Milan Jovanović Batut” [30].

The number of confirmed SARS-CoV-2-positive individuals and total number of deaths from COVID-19 infection are calculated as a sum of daily reported positive cases and daily reported deaths and are presented as a total value per calendar month.

Confirmation of a positive case of the SARS-CoV-2 virus at the beginning of the pandemic (2020) was based exclusively on the results of the RT-PCR test. However, from mid-2021, the WHO allowed the use of validated antigen tests to report positive cases, so both methods (RT-PCR + antigen test) were used here to test for positivity. In text, this is outlined as TOTAL CASES (for a specific period).

Deaths from COVID-19 infection were considered in patients who died under the diagnosis of UO7.1 (COVID-19, virus identified), according to the ICD classification of diseases (patients died “from” COVID-19 infection). In text, this is outlined as TOTAL DEATHS (for a specific period).

The COVID-19-positive patients who died from other causes have not been included in this study (patients died “with” COVID-19 infection).

4. Public health measures (periods of social distancing and vaccination period)—the Ministry of Health of the Republic of Serbia [29], Statistical Office of the Republic of Serbia [31]. Periods of increased social distancing included 10 months divided into four periods:

- Period 1 (March–May 2020: official lockdown period, state of emergency),
- Period 2 (July–August 2020: stricter measures),
- Period 3 (November 2020–January 2021: partial closures),
- Period 4 (March–April 2021: “mini” lockdown period).

The influence of increased social measures (10 months) was analyzed compared to the months without increased social measures (26 months).

Vaccination rates were presented as the number of totally administered doses of every type of vaccine during the period of extensive vaccination campaign in the RS (24 December 2020–23 June 2022). The influence of the vaccination campaign (19 months) was analyzed compared to the months without the vaccination campaign (17 months).

All datasets were harmonized to a monthly level from March 2020 to March 2023.

### 2.3. Statistical Analysis

The results are expressed via descriptive statistics, i.e., measures of a central tendency (mean, $\bar{x}$ ± standard deviation, SD). SPSS software v20 was used. After confirming the normal distribution, the means of the two groups were tested by a two-sample Student *t*-test for independent variables. In cases of >2 groups, a one-way ANOVA on ranks was used (with the Tukey post hoc analysis). Pearson’s product moment correlation coefficient (r) was used to test the correlation between two independent parameters (0.5 > r ≥ 0.3 indicated moderate positive linear correlation; r ≥ 0.5 indicated strong positive correlation, whereas −0.5 < r < −0.3 indicated moderate negative linear correlation; and r ≤ −0.5 indicated strong negative correlation). Simple linear regression analysis was used to test if the vaccination rates (expressed as the total number of administered doses) affected the TOTAL CASES and TOTAL DEATHS from COVID-19 infection. The relationship between the dependent and independent variables was confirmed by a multiple linear regression. The Durbin–Watson (DW) test was used to check for the autocorrelation of residuals and the variance inflation factor (VIF) was used to check for multicollinearity among independent variables. VIF was calculated as follows: VIF = 1/(1 − R^2^), where R is the coefficient of determination of the regression analysis. VIF > 5 was suggestive of multicollinearity. A DW value equal to or near 2 suggested no autocorrelation, whilst a DW > 2 meant negative autocorrelation and a DW < 2 meant positive autocorrelation. In the case of positive autocorrelations, the correction was made with the generalized least squares with autoregressive errors (GLSAR) method. For the comparison of the two independent variables, a non-parametric Mann–Whitney rank sum test was used. Probability values (*p*) < 0.05 were considered statistically significant, while *p*-values < 0.01 were considered highly statistically significant.

## 3. Results

### 3.1. Air Quality Index (AQI) and the Concentrations of Pollutants in the Air

**Figure 1:**
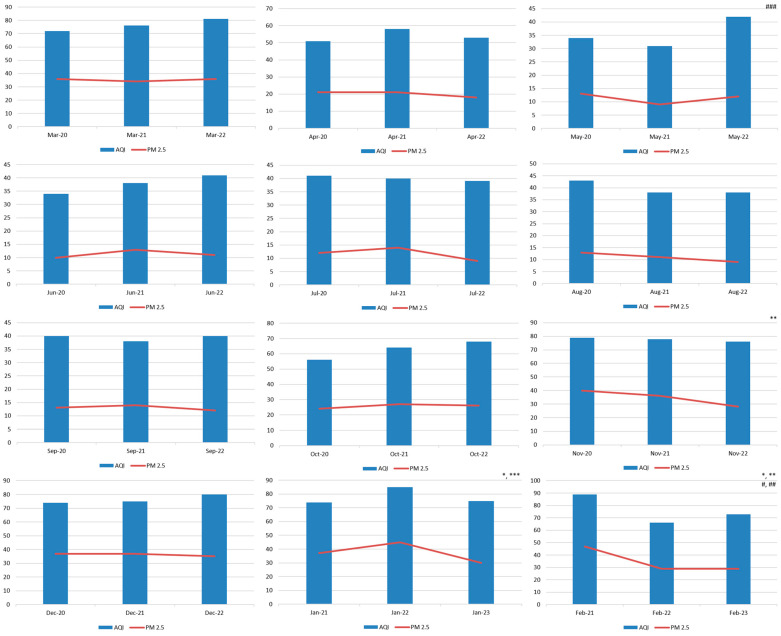
The trend of AQI values and PM_2.5_ concentration according to specific calendar months. There were no statistically significant differences in terms of AQI values, except for May and February. There is a statistically significant decrease in PM_2.5_ concentrations, which was the most pronounced during the winter months (November, January, and February). Statistical significance (*p* < 0.05) is shown as follows: for the PM_2.5_ (*—Δ2021/2020, **—Δ2022/2020, and ***—Δ2022/202); (Δ2023/2022 for January and February); for the AQI (^#^—Δ2021/2020, ^##^—Δ2022/2020, and ^###^—Δ2022/2021 (Δ2023/2022 for January and February).

The average values of AQI and concentrations of PM_2.5_ in the RS during study periods I, II, and III are presented in Table 1. There has been a statistically significant decrease in the PM_2.5_ concentrations in period III compared to period I (*p* = 0.03). The average values of the AQI and PM_2.5_ and their trend according to specific calendar months over the study years are shown in Figure 1.

The concentrations of other (specific) pollutants in the air are presented in Table 2. Concentrations are shown during the calendar year. Mean annual concentrations of major air pollutants (SO_2_, NO_2_, and PM_10_) and heavy metals in the air (Pb, As, Cd, and Ni) are presented in Table 3. The data also present the values of the extremes. According to this, annual air concentrations above upper allowed limits were noted mostly for As, while the most favorable values were noted for Ni. There were no statistically significant differences in the mean annual concentrations of air pollutants or heavy metals between the study periods, except for the concentrations of PM_10_ that showed a significant decrease in period III. There was no statistically significant correlation between the number of total cases and the concentrations of specific air pollutants. There were strong positive correlations between the total deaths from COVID-19 infection and the concentration of Pb (r = 0.7, *p* = 0.002), As (r = 0.6, *p* = 0.04), and Cd (r = 0.7, *p* = 0.02) in period III.

### 3.2. Average Air Temperatures (AIR TEMP) and Precipitation Rates (PREC)

**Figure 2:**
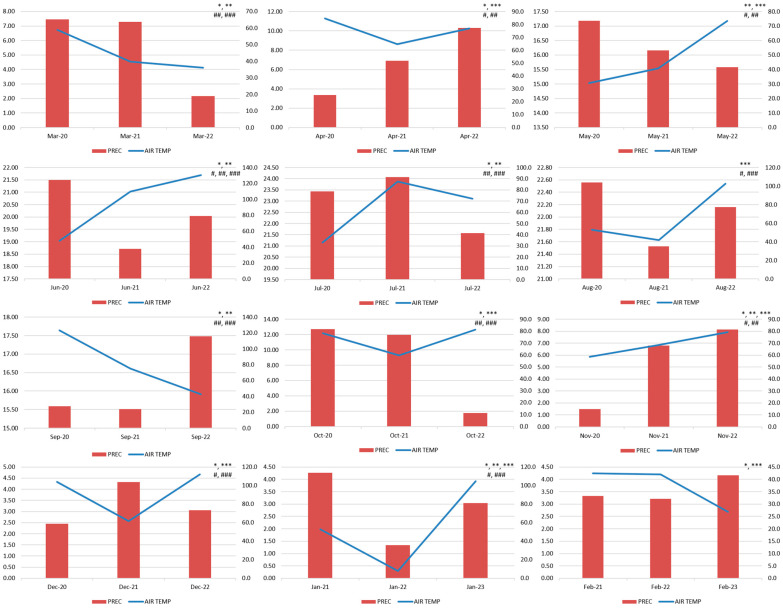
The average values of AIR TEMP and PREC according to specific calendar months over the years. Although no statistically significant difference was observed comparing the three sub-periods of the study, clear differences are seen in the specific calendar months. Statistical significance (*p* < 0.05): AIR TEMP (*—Δ2021/2020, **—Δ2022/2020, and ***—Δ2022/2021 (Δ2023/2022 for January and February)); PREC (^#^—Δ2021/2020, ^##^—Δ2022/2020, and ^###^—Δ2022/2021 (Δ2023/2022 for January and February)).

The average values of AIR TEMP and PREC in the RS during periods I, II, and III are presented in Table 3. There were no statistically significant differences in the AIR TEMP and PREC between the study periods. The average values of the AIR TEMP and PREC and their trend according to specific calendar months over the years are shown in Figure 2.

**Table 3:** Average air temperatures and precipitation rates in the RS during the three study sub-periods.

Period/Climate Factors	I	II	III	ΔII/I	ΔIII/II	ΔIII/I
AIR TEMP (°C, $\bar{x}$ ± SD)	11.8 ± 7.1	11.2 ± 8.1	12.2 ± 7.7	*p* > 0.05	*p* > 0.05	*p* > 0.05
PREC (mm, $\bar{x}$ ± SD)	66.8 ± 36.3	56.1 ± 25.2	61.7 ± 30.4	*p* > 0.05	*p* > 0.05	*p* > 0.05

### 3.3. The Consequences of the COVID-19 Pandemic in the RS—Total Cases and Total Deaths

The total number of cases in periods I, II, and III were 458,859, 1,421,717, and 582,723, respectively, for a total of 2,463,299 cases. The total number of deaths in periods I, II, and III were 4443, 10,798, and 2816, respectively, for a total of 18,057 deaths (this data was last updated on the 13 April 2024). The average number of cases and deaths from COVID-19 infection in the RS during periods I, II, and III are presented in Table 4. There was a statistically significant increase in the average number of reported cases in period II compared to period I. As for the average number of deaths from COVID-19 infection, a statistically significant increase was noted in period II, whereas a statistically significant decrease was noted in period III (see Table 4).

### 3.4. The Influence of the Environmental and Climate Factors on the Number of Total Cases and Total Deaths

**Figure 3:**
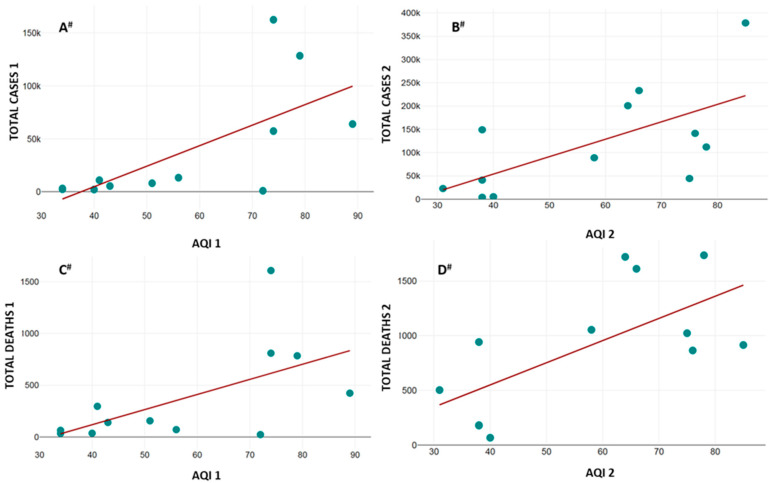
Correlation between AQI and TOTAL CASES (**A**,**B**) and TOTAL DEATHS (**C**,**D**) from COVID-19 infection. X axis—average values of AQI for study periods I and II; Y axis—TOTAL CASES (**A**,**B**) and TOTAL DEATHS (**C**,**D**) from COVID-19 infection for study periods I and II. AQI 1 (average values of AQI during period I), AQI 2 (average values of AQI during period II), TOTAL CASES 1 (total number of cases during period I), TOTAL CASES 2 (total number of cases during period II), TOTAL DEATHS 1 (total number of deaths from COVID-19 infection during period I), and TOTAL DEATHS 2 (total number of deaths from COVID-19 infection during period II). Correlation direction and intensity are represented as **^#^**—strong positive (all variables).

**Figure 4:**
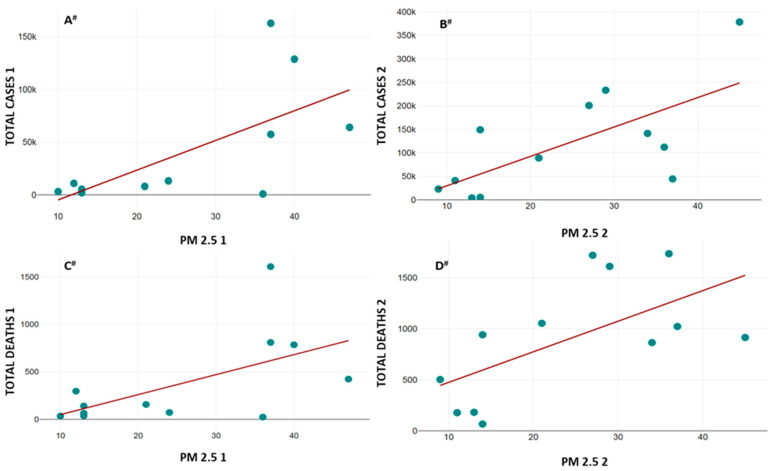
Correlation between the average values of PM_2.5_ and TOTAL CASES (**A**,**B**) and TOTAL DEATHS (**C**,**D**) from COVID-19 infection. X axis—average PM_2.5_ concentrations for study periods I and II; Y axis—TOTAL CASES (**A**,**B**) and TOTAL DEATHS (**C**,**D**) from COVID-19 infection for study periods I and II. PM2.5 1 (average values of AQI during period I), PM2.5 2 (average values of AQI during period II), TOTAL CASES 1 (total number of cases during period I), TOTAL CASES 2 (total number of cases during period II), TOTAL DEATHS 1 (total number of deaths from COVID-19 infection during period I), and TOTAL DEATHS 2 (total number of deaths from COVID-19 infection during period II). Correlation direction and intensity are represented as **^#^**—strong positive (all variables).

**Figure 5:**
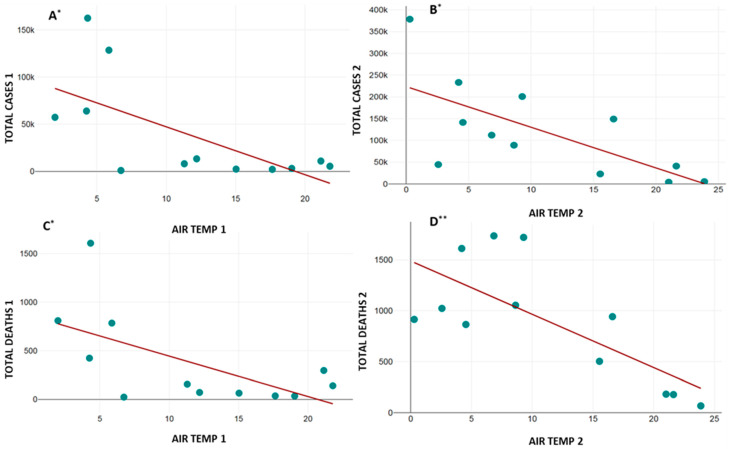
Correlation between average air temperatures (AIR TEMP) and total cases (**A**,**B**) and total deaths (**C**,**D**) from COVID-19 infection. X axis—average AIR TEMP for study periods I and II; Y axis—total cases (**A**,**B**) and total deaths (**C**,**D**) from COVID-19 infection for study periods I and II. AIR TEMP 1 (average AIR TEMP during period I), AIR TEMP 2 (average AQI during period II), TOTAL CASES 1 (total number of cases during period I), TOTAL CASES 2 (total number of cases during period II), TOTAL DEATHS 1 (total number of deaths from COVID-19 infection during period I), TOTAL DEATHS 2 (total number of deaths from COVID-19 infection during period II). Correlation direction and intensity are represented as: *—strong negative and **—very strong negative.

The correlation of the average values of the AQI, PM_2.5_, AIR TEMP, and PREC with TOTAL CASES and TOTAL DEATHS from COVID-19 infection is presented in Table 5. The data from the abovementioned correlations that showed statistical significance are graphically presented in Figure 3, Figure 4 and Figure 5.

As for the AQI, strong positive correlation was noted in periods I and II for TOTAL CASES (Figure 3A,B) and TOTAL DEATHS from COVID-19 infection (Figure 3C,D).

As for the concentrations of PM_2.5_, strong positive correlation was noted in periods I and II for TOTAL CASES (Figure 4A,B) and TOTAL DEATHS from COVID-19 infection (Figure 4C,D).

As for the AIR TEMP, strong negative correlation was noted for TOTAL CASES in periods I and II (Figure 5A,B). Strong negative correlation in period I (Figure 5C) and very strong negative correlation in period II (Figure 5D) were noted for TOTAL DEATHS from COVID-19 infection.

There were no correlations between the PREC and TOTAL CASES and TOTAL DEATHS from COVID-19 infection during the study period.

There were no correlations in period III.

### 3.5. Testing for Autocorrelation and Multicolinearity Among Independent Variables

In order to determine whether there is autocorrelation or multilinearity between the independent variables, we applied the Durbin–Watson test and calculated the VIF (variance inflation factor). Regarding the correlation of ecoclimatic factors and the total number of new cases, the DW value was 1.16 (R^2^ = 0.19), suggesting a positive autocorrelation among the variables. After GLSAR corrections, DW values were ~2 (R^2^ = 0.15), avoiding the autocorrelation. In this corrected model none of the independent variables (AQI, AIR TEMP, and PREC) influenced the TOTAL CASES, probably due to the presence of multicollinearity among variables which was observed for AQI (VIF = 5.7) and AIR TEMP (VIF = 5.6) as opposed to PREC (VIF = 1.1). Regarding the correlation of ecoclimatic factors and the TOTAL DEATHS from COVID-19 infection, the DW value was 0.83 (R^2^ = 0.26), suggesting a strong positive autocorrelation among the variables. After GLSAR corrections, DW values were ~1.8 (R^2^ = 0.15), avoiding the autocorrelation. In this corrected model, TOTAL DEATHS from COVID-19 infection was inversely correlated with the average AIR TEMP (*p* = 0.05). There was no multicollinearity between AQI, AIR TEMP, and PREC (VIF = 3.3, 2.7, and 4.9, respectively).

### 3.6. The Influence of Public Health Measures on the Total Cases and Total Deaths from COVID-19 Pandemic in the RS

**Figure 6:**
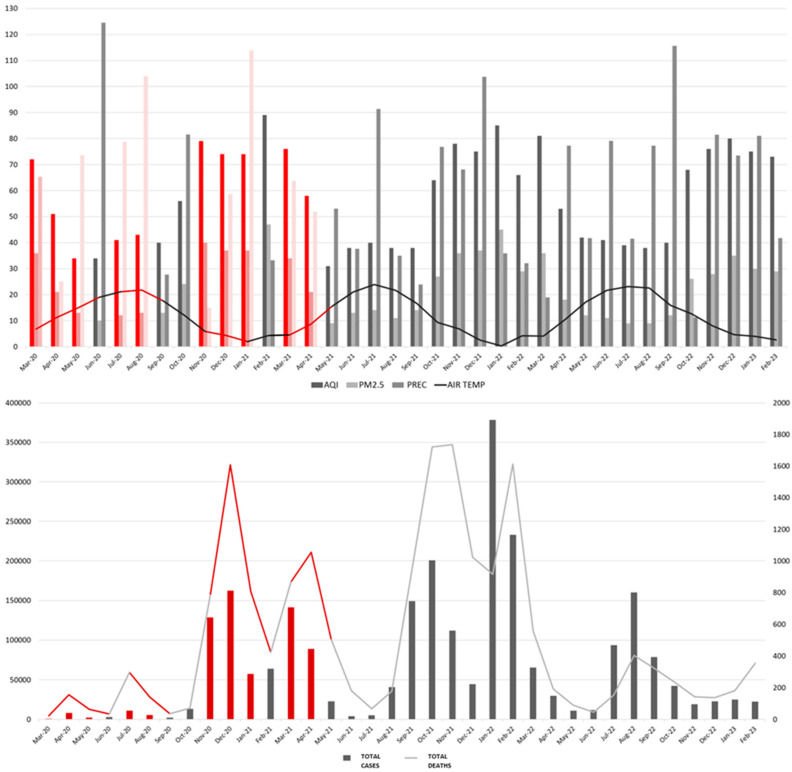
The relationship between the four periods of increased social measures and the AQI, PM_2.5_, PREC, AIR TEMP (**top**), and TOTAL CASES, and TOTAL DEATHS (**bottom**) from COVID-19 infection. The characteristics of the four periods (10 months in total) are, from left to right: First (March–May 2020): the official lockdown (state of emergency), with a curfew, schools, cafés, and public transport closure, and a movement ban for the elderly. Second (July–August 2020): stricter measures (second wave), with the gathering ban (10+ people), mandatory face masks, and limited working hours. Third (November 2020–January 2021): partial closures (third wave) with reduced working hours, cafés/restaurants closure from 9 p.m., and online schools. Fourth (March–April 2021): a mini lockdown period, where all facilities except grocery stores, pharmacies, and gas stations were closed. Partial measures (weekend closures) were present until the end of April 2021, with the closures of catering facilities on weekends, while shops and pharmacies were allowed to operate. Data during the four periods are distinguished by red lines/columns.

**Figure 7:**
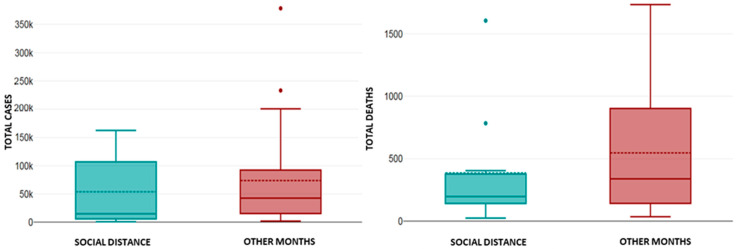
The number of TOTAL CASES (**left**) and TOTAL DEATHS (**right**) from COVID-19 infection during the months of enhanced social distance and other months. The following outliers were identified: TOTAL CASES (OTHER MONTHS): January 2022: 378,459 and February 2022: 233,177; and TOTAL DEATHS (SOCIAL DISTANCE): December 2020: 1607 and March 2021: 865. There were no statistically significant differences in the number of TOTAL CASES and TOTAL DEATHS from COVID-19 infection during the compared periods.

**Figure 8:**
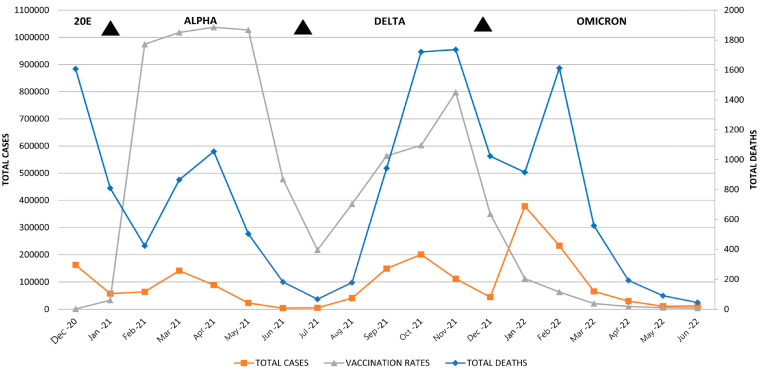
An overview of the vaccination period in the RS (December 2020–June 2022), marked as the red scattered area, and its relation to the total cases and total deaths from COVID-19 infection per study month. The upper part of the figure shows the time periods in which different variants of the SARS-CoV2 virus were present in the RS: variant 20E (October 2020–January 2021), variant 20I (Alpha) (February–July 2021), variant 21J (Delta) (July–December 2021), and variant 21K (Omicron BA.1) (December 2021–June 2022). The figure shows that the first half of 2021 was the most intensive vaccination period, when the mass immunization of the population was undertaken.

**Figure 9:**
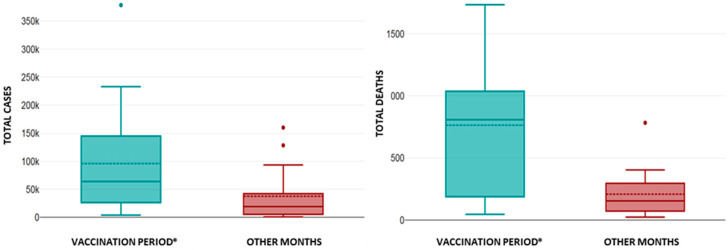
The influence of the vaccination period on TOTAL CASES (**left**) and TOTAL DEATHS (**right**) from COVID-19 infection. Several outliers were identified: TOTAL CASES (VACCINATION PERIOD): Jan 2022: 378,459, and TOTAL CASES (OTHER MONTHS): November 2020: 128,484, and August 2020: 160,170. TOTAL DEATHS from COVID-19 infection (OTHER MONTHS): November 2020: 784. There was a statistically significant higher number of TOTAL CASES and TOTAL DEATHS from COVID-19 infection during the VACCINATION PERIOD (* *p* < 0.02).

Considering the detected multicollinearity between the variables, in this section we aim to determine the impact of public health measures for a better conclusiveness of the obtained results. Firstly, we compared the effects of social distancing measures (official lockdown(s), the ban on gatherings, and mandatory mask wearing) implemented over 10 months with the periods during which these measures were not implemented (Figure 6 and Figure 7). Secondly, we compared the vaccination periods over a course of 19 months with the months during which vaccination was not implemented (Figure 8 and Figure 9).

#### 3.6.1. Months of Increased Social Distancing

The average values of AQI, PM_2.5_, TEMP, and PREC during the months of increased social distancing were 60.2 ± 16.8, 26.4 ± 11.4 µg/m^3^, 10.1 ± 7 °C, and 65 ± 30.7 mm; meanwhile, in other months the average values were 56.8 ± 19.1, 22.4 ± 11.9 µg/m^3^, 12.3 ± 7.6 °C, and 60.1 ± 30.7 mm, respectively. There were no statistical differences in the average values of AQI, PM_2.5_, TEMP, and PREC during the months of increased social distancing compared to other months (*p* > 0.05 for all). The total number of cases and deaths from COVID-19 infection during the months of increased social distancing was 606,537 and 5798, while in other months it was 1,856,762 and 12,259, respectively. The average number of cases and deaths from COVID-19 infection during the months of increased social distancing were 60,653 ± 64,568 and 579 ± 525, while in other months it was 71,413 ± 89,100 and 471 ± 528, respectively. There have been no statistically significant differences in the average number of cases and the average number of deaths from COVID-19 infection between the months of increased social distancing compared to other months (*p* > 0.05 for both). Boxplots showing the TOTAL CASES and TOTAL DEATHS from COVID-19 infection during the months of increased social distancing compared to other months are presented in Figure 7.

#### 3.6.2. Vaccination Period

The vaccination in the RS started on 24 December 2020 and lasted until 23 June 2022. The relationship between this period and the burden of the COVID-19 pandemic in the RS is presented in Figure 9. The average values of AQI, PM_2.5_, AIR TEMP, and PREC during the vaccination period were 60 ± 19.1, 25.7 ± 12.7 µg/m^3^, 10.5 ± 7.8 °C, and 57.7 ± 27.2 mm; meanwhile, in other months the average values were 55.2 ± 17.7, 21.1 ± 10.5 µg/m^3^, 13.2 ± 7 °C, and 65.8 ± 33.9 mm, respectively. There were no statistical differences in the average values of AQI, PM_2.5_, AIR TEMP, and PREC during the vaccination period compared to other months (*p* > 0.05 for all). The TOTAL CASES and TOTAL DEATHS from COVID-19 infection during the vaccination period were 1,823,966 and 14,522, while in other months those respective values were 639,333 and 3535, respectively. The average number of cases and deaths from COVID-19 infection during the vaccination period were 95,999 ± 96,884 and 764 ± 586, while in other months the respected average values were 37,608 ± 48,274 and 207 ± 188, respectively. There were statistically significant higher numbers of TOTAL CASES and TOTAL DEATHS from COVID-19 infection in the vaccination period (*p* < 0.02 for both). Boxplots showing the number TOTAL CASES and TOTAL DEATHS from COVID-19 infection during the vaccination period compared to other months are presented in Figure 9.

##### The Influence of Vaccination Rates on Total Cases and Total Deaths from COVID-19 Infection

As of 23 June 2022, the RS had administered a total of 8,534,685 COVID-19 vaccine doses. According to the official data, by this date RS had administered 3,354,075 first doses, 3,278,198 second doses, and 1,902,412 third (booster) doses of COVID-19 vaccines. Regarding the total population of the RS, this means that 48% of the population received the first dose, 47% received the second dose, and 27% received the third dose. All four types of vaccines were available: Sinopharm^®^, Pfizer/BioNTech^®^, Sputnik V^®^, and AstraZeneca^®^. The largest number of vaccinated people (62.2%) received the Sinopharm^®^ vaccine (3.4 million doses). There was no correlation between the total number of vaccine doses and the TOTAL CASES and TOTAL DEATHS from COVID-19 infection (*p* > 0.05).

## 4. Discussion

This paper for the first time examined the influence of ecoclimatic factors (air quality, average air temperatures, and precipitation rates) on the total number of cases and deaths from COVID-19 infection in the RS. The study covered a period of three years, using data from publicly available official sources. Interesting but moderately conclusive results were obtained, which is why the impact of additional factors, such as public health measures (periods of social distancing and vaccination period), is also covered.

For years the RS has been among the countries with the worst air quality, as confirmed by numerous official reports. According to one of them, three out of the fifteen most polluted regional European cities in 2020 were from the RS [32]. Moreover, nearly half of the total of the RS’s population live in areas where the annual PM_2.5_ levels exceed the WHO Air Quality Guideline (25 µg/m^3^), which is among the highest in Europe [33]. This is understandable considering numerous environmental, urban, and infrastructural issues in the RS, which are related to the energy and transport sectors, waste dump sites, and industrial activities (petrochemical industry, chemical plants, metallurgical complexes, and thermal power plants). Particularly big problems are mini hydropower plants and deforestation [34]. Its capital, Belgrade, is very often among the most polluted capitals in the world, probably due to dense road traffic and proximity of the coal-fired power plant and industrial plants. During the course of our study, AQI and concentrations of PM_2.5_ were in accordance with the average annual values of AQI (52, 58, 61, and 50) and concentrations of PM_2.5_ (21, 25, 24, and 17) in the RS during 2020, 2021, 2022, and 2023, respectively [24,25,26,27]. This is classified as “moderately unhealthy” by the AQI-US standards. Overall, the values of the AQI values are way higher than the EU average, comparable with Bulgaria, Latvia, and Poland, but certainly lower than surrounding countries, like North Macedonia and Bosnia and Herzegovina [35]. Interestingly, apart from the concentrations of PM_10_ there were no statistically significant differences in the concentrations of other polluting substances in the air (heavy metals and oxides) during the observed periods, neglecting their influence on the air quality in the RS. Concentrations of PM_10_, however, showed statistically significant reductions over the years. This could be explained due to several reasons: 1° increase in the number of measurement sites in 2023 compared to 2020 (from 33 to 68, as mentioned in Table 2), which enabled more precise monitoring and identification of pollution sources; 2° reduction in the number of days with exceeding limit values (decreased by 19% in 2023); or 3° reduction in the number of cities with excessive pollution (in 2023 the air was excessively polluted in 21 cities, which is fewer than in previous years). However, PM_10_ is less correlated to the severity of COVID-19 infection than PM_2.5_ because PM_10_ particles do not penetrate as deeply into the lungs as PM_2.5_. Also, the increase in PM_2.5_ of just 1 µg/m^3^ is associated with a 15% higher mortality rate from COVID-19 infection [9], which prolonged hospitalization courses, as confirmed by the studies from Italy, China and the USA [36,37,38].

Poor air quality is an etiological factor for numerous diseases, including infectious diseases transmitted by droplets, such as COVID-19 infection. On the one hand, pollutants presented in the air may enhance the degree of viral transmission; while on the other hand, they may act synergistically with the virus, leading to a more serious clinical outcome [39]. Our study showed a clear, positive correlation between the AQI and the total number of cases and the total number of deaths from COVID-19 infection, which is consistent with the data from the literature [40]. This impact was more intense during the second period of the study, which coincides with the peak periods of the pandemic. Increased mortality rates from COVID-19 infection in conditions of long-term exposure to poor air quality may be seen in a wide number of national reports (for example, Indonesia, Denmark, and Brazil) [41,42,43]. Overall, our results showed that AQI was associated with the peak of total cases, even though there was no decrease in the concentrations of specific air pollutants during the COVID-19 pandemic.

Global consequences of climate change are affecting the RS as well. For example, the year 2020 has been recorded as the seventh-driest year since 1951 [19]. The mean annual air temperature (11.9 °C) showed that nine out of the ten warmest years in the RS for a period of 69 years (1951–2020) were detected in the last two decades. Although the summer of 2020 was the second-wettest summer in the RS in the last 70 years, it was higher than the average (per corresponding month) in March 2020, when the COVID-19 pandemic started, especially in the southeastern and southern parts of the country. In contrast, April 2020 was the sixth-driest April since 1951, with heat waves detected in some parts. From May to August, some cities in the southeast region of the RS experienced the wettest days, with the precipitation rates above average, even in mountainous regions. On the other hand, the upcoming fall was the warmest and driest, setting up records in several cities. Even in November and December, heat waves were detected at several weather stations. Therefore, the southern, southeastern, and southwestern regions of the RS were extremely humid. Similar meteorological patterns have been observed through 2021, with temperature and precipitation extremes mostly observed in the winter [20]. This period (November 2020–January 2021) has been recorded as the wettest three months since 1951. However, there were notable differences between the summer and winter of 2020 and those of 2021, as July 2021 was hotter (+3 °C) compared to July 2020, and December 2021 was colder (−2 °C) compared to December 2020 [19,20]. According to our results, in both those months the total number of COVID-19 cases was around two times lower. With an average air temperature of 12.1 °C, 2022 was the second-warmest year since 1951 [21]. Compared with the previous period, December was noted as the second warmest, with several heat waves and a +4 °C average air temperature. Furthermore, January 2023 was the sixth-wettest and second-warmest January since 1951, with the highest daily temperature of 20 °C, measured on the 1st of January [22]. Therefore, air temperature and precipitation rate perturbations were highly prominent during the COVID-19 pandemic, with many extremes compared to the pre-pandemic period. This is also illustrated on our graph, where the trend of increasing average air temperatures in the winter months (November–January) is most clearly observed.

At the beginning of the COVID-19 pandemic, Sobral et al. [44] and Pequeno et al. [45] reported a negative correlation between the average air temperatures and the total number of reported cases, which was confirmed by a study from Italy [46]. Similarly, in the USA an increase in the average temperature of 1% decreased the number of reported cases by 0.002% [47]. According to this paper, American states with lower air temperatures had a significantly higher number of total cases and deaths from COVID-19 infection compared to the countries with higher average air temperatures. In contrast, higher average air temperatures were associated with an increase in the total number of deaths from COVID-19 infection in some European [48] and Asian [49] countries.

Countries with higher precipitation rates (frequent rainfalls) had notably high viral transmission, so a strong positive correlation with the number of reported cases was observed [50]. As for studies regarding temperature and precipitation correlations, a significantly greater number of COVID-19-related deaths (up to four times) was observed in places with average temperatures of 4–12 °C and a relative humidity of 60–80% [51]. We did not find a correlation between total cases and total deaths and the precipitation rates, although there were significant changes in the corresponding months over the years, whereas average humidity has not been considered.

The COVID-19 pandemic showed its brighter side by reducing global air pollution [52,53,54]. The reduction in fossil fuel consumption caused by lockdowns around the world significantly improved air quality by 65% in cities worldwide, whereas 84% of the countries experienced an overall improvement [55]. The capital of the RS, Belgrade, however, has continued to be among the most polluted cities worldwide during the pandemic [32]. Our results indicated that there was no significant improvement in the air quality during the period of increased social distancing, which is in contrast to other reports [56,57,58,59]. However, our methodology implied the collision of all periods during which social distancing measures were in force (a total of 10 months). This means that we have combined the period of official and exclusive lockdown (state of emergency) and periods of enhanced social measures but outside the official lockdown period. When only the lockdown period (March–May 2020) is observed the effect on the reduction in AQI is clearly visible (this result is not shown), so it can be said that we have also confirmed this relationship. This is in line with other national studies [60,61] and may be due to the strictest measures in the fight against COVID-19 pandemic compared to other countries in the region and in Europe [62].

In mid-December 2020, the unfavorable epidemiological situation forced the authorities in the RS to start the intensive vaccine procurement, so after the United Kingdom and Switzerland, the RS became the third country in Europe to start vaccinations [63,64]. Shortly after the Prime Minister had received the first vaccine in late December, massive immunization in the RS began and lasted for the next 19 months. Despite its availability on the market at the beginning of 2021, the pandemic reached its peak a year after the first case [4]. This initiated authorities to undertake national campaigns for mass immunization among citizens, heralding the second phase of the COVID-19 epidemic in the RS. As a result, by April 6, 2021, more than 75 000 people were vaccinated [29]. By the time the delta variant of the virus had spread throughout the country, vaccination rates in the RS appeared to reach a plateau that corresponded to the average of 73% of the total EU population (first dose) [63]. During the following months, the vaccination rates were satisfactory, as 22.8% of the population received at least one dose, and 16.7% of the population were fully vaccinated by the middle of 2021 [29]. Soon afterwards, effective vaccination in the RS resulted in a decreased slope of COVID-19 related deaths (see July 2021, Table 4). Unfortunately, after the initial mass vaccination, the number of deaths significantly increased, probably as a consequence of the new viral variant (delta). The highest observed mortality during the pandemic was in October–November 2021. During the following year, the pandemic entered a slower phase, and the vaccination rates were stable, with 23 June 2022 being the last follow-up date, according to world statistics [1]. During 2021, two important peaks regarding mortality occurred, i.e., April and November, but it is also important to notice the abrupt increase in vaccine dose administration in February and its high plateau until May, which resulted in lower mortality in the upcoming months (June–August), after which the delta variant was more prominent. However, this diagram indicates the favorable results (at least short-term) of a country’s quick response to the COVID-19 pandemic and efficacious vaccine campaign [65]. During the vaccination period, a statistically significantly higher numbers of cases and deaths from COVID-19 infection were observed, but this is not a sample–effect relationship and is explained by the fact that the vaccination was carried out during the most intense period of the COVID-19 pandemic. Correlation between the total number of given vaccine doses and the total cases or total deaths was not found, contrary to another study [66].

The limitations of this study have to be addressed. First, environmental and health-related data were collected from different sources, such as publicly available international/national websites, annual reports, health statistics, and articles written by official authorities. Although those sources are official and validated, we did not have a unique dataset. Second, we could not reach for the precise monthly data on the total number of deaths from COVID-19 infection for the specific cities of the RS as to make a proper statistical correlation between environmental factors and COVID-19-related deaths in specific regions of a country. Therefore, we were unable to examine potential differences between different geographical areas in the RS. These facts could be important for a more comprehensive understanding and strong correlation of the environmental factors and the course and the number of deaths from COVID-19 infection in the RS. The third issue was not addressing all confounding variables that could have impacted COVID-19-related deaths, like the presence of comorbidity or the exact impact of habits and lifestyle on the unfavorable outcome from COVID-19 infection. For example, we did not cover the percentage of people with underlying chronic non-communicable diseases (or risk factors) for whom COVID-19 infection was the direct cause of death and therefore affected mortality from COVID-19 infection. The outcome of COVID-19 infection is influenced by many things, not only environmental but also demographic and social factors; thus, a clear correlation would require collaboration with different clinical centers, access to their local data, the inclusion of all confounding variables, more extensive statistical analyses, and a methodological approach [67,68,69,70]. The influence of individual risk factors will be the subject of our next research.

## 5. Conclusions

Here we wanted to determine whether the relatively high number of deaths from COVID-19 infection in the RS, despite a stable public health policy, technical–organizational capacities, and a quick and well-founded response of the state, was influenced by specific external factors such as ecological and climatic factors.

For the total number of cases and deaths from COVID-19 infection, a strong, positive correlation was found with the air quality and a strong, negative correlation with the average air temperatures. However, a certain degree of multicollinearity among the variables was observed, which is why the effects of vaccinations and the period of enhanced public health measures were also considered. During the vaccination period a higher number of total cases and total deaths from COVID-19 infection were determined, but this cannot be attributed to a causative relationship. In order to examine other influences on the burden of the COVID-19 pandemic in the RS, our further research will employ the following two directions: examining the influence of individual risk factors (pre-existing non-communicable diseases (cardiovascular, respiratory, and malignant), habits, physical activity, and pharmacotherapy) and spatiotemporal differences between the cities in the RS. This would cover all aspects of a global threat like the COVID-19 pandemic and prepare the country for more effective measures in case of potentially similar epidemiological scenarios.

## Figures and Tables

**Table 1:** Average values of air quality index (AQI) and concentrations of fine particulate matters 2.5 µm (PM_2.5_) in the air during the three study sub-periods.

Period/Air Quality	I	II	III	ΔII/I	ΔIII/II	ΔIII/I
AQI ($\bar{x}$ ± SD)	57.2 ± 19.4	57.2 ± 19.2	58.8 ± 18.1	*p* > 0.05	*p* > 0.05	*p* > 0.05
PM_2.5_ (µg/m^3^, $\bar{x}$ ± SD)	25.2 ± 13.3	24.2 ± 12.1	21.2 ± 10.4	*p* > 0.05	*p* > 0.05	*p* = 0.03

**Table 2:** The average concentrations of major air pollutants (Pb, As, Cd, Ni, SO_2_, NO_2_, and PM10). ^#^—Δ 2023/2022 and ^##^—Δ 2023/2020.

Air Pollutants	Period	Mean Annual Values ( $\bar{x}$ ± SD) (min–max)	Upper Annual Limit	Measuring Stations (n)	Measuring Stations that Exceeded the Upper Limit (n)	*p*
Pb (ng/m^3^)	2020	62 ± 136 (2–651)	500	28	1	>0.05
	2021	35.7 ± 81.3 (3–331)		16	0	
	2022	19.8 ± 40.4 (5–165)		15	0	
	2023	13.3 ± 26.6 (3–112)		16	0	
As (ng/m^3^)	2020	6.7 ± 16.2 (0.01–62)	6	24	2	>0.05
	2021	11.4 ± 29.2 (0.7–132)		22	5	
	2022	5 ± 11.2 (1–50)		19	4	
	2023	4.1 ± 8.4 (0.3–40)		27	4	
Cd (ng/m^3^)	2020	1.2 ± 1.7 (0.003–6)	5	24	1	>0.05
	2021	0.7 ± 1.4 (0.1–6.4)		22	1	
	2022	0.6 ± 1.1 (0.2–5)		19	0	
	2023	1.1 ± 2.4 (0.1–10)		27	2	
Ni (ng/m^3^)	2020	3.9 ± 2 (0.01–8)	20	24	0	>0.05
	2021	5 ± 4.6 (1–20)		22	0	
	2022	3.5 ± 1.4 (1–7)		19	0	
	2023	3.1 ± 2.1 (1–9)		27	0	
SO_2_ (µg/m^3^)	2020	16.4 ± 13.2 (6–74)	50	40	2	>0.05
	2021	14 ± 7.1 (5–44)		37	1	
	2022	13.1 ± 5 (5–31)		45	0	
	2023	12.3 ± 3.7 (6–24)		56	0	
NO_2_ (µg/m^3^)	2020	20.2 ± 7.7 (8–38)	40	39	0	>0.05
	2021	22.1 ± 10.3 (3–57)		39	2	
	2022	22.2 ± 9.4 (3–54)		50	1	
	2023	20.7 ± 8.5 (3–49)		63	2	
PM_10_ (µg/m^3^)	2020	39.1 ± 11.2 (17–63)	50	33	5	0.001 ^#,##^
	2021	34.9 ± 8.9 (22–64)		35	3	
	2022	36.1 ± 7.9 (17–67)		53	2	
	2023	32 ± 7.4 (14–60)		68	1	

**Table 4:** The average number of cases and deaths from COVID-19 infection in the RS during the three study sub-periods.

Period/COVID-19	I	II	III	ΔII/I	ΔIII/II	ΔIII/I
Cases ($\bar{x}$ ± SD)	38,238 ± 54,906	118,476 ± 111,345	48,560 ± 44,277	*p* = 0.03	*p* > 0.05	*p* > 0.05
Deaths ($\bar{x}$ ± SD)	370 ± 480	900 ± 588	235 ± 148	*p* = 0.01	*p* = 0.002	*p* > 0.05

**Table 5:** The correlation between mean annual values of air temperatures (AIR TEMP), precipitation rates (PREC), AQI, and PM_2.5_ and (A) TOTAL CASES and (B) TOTAL DEATHS from COVID-19 infection in the defined period. Correlation direction and intensity are represented as *—strong negative; **—very strong negative; and ^#^—strong positive.

A. TOTAL CASES	I (March 2020–March 2021)	II (March 2021–March 2022)	III (March 2022–March 2023)
AQI	(r = 0.69, *p* = 0.01) ^#^	(r = 0.65, *p* = 0.02) ^#^	*p* > 0.05
PM_2.5_ (µg/m^3^)	(r = 0.68, *p* = 0.01) ^#^	(r = 0.68, *p* = 0.01) ^#^	*p* > 0.05
AIR TEMP (°C)	(r = −0.66, *p* = 0.02) *	(r = −0.68, *p* = 0.01) *	*p* > 0.05
PREC (mm)	*p* > 0.05	*p* > 0.05	*p* > 0.05
B. TOTAL DEATHS	I (March 2020–March 2021)	II (March 2021–March 2022)	III (March 2022–March 2023)
AQI	(r = 0.59, *p* = 0.04) ^#^	(r = 0.66, *p* = 0.02) ^#^	*p* > 0.05
PM_2.5_ (µg/m^3^)	(r = 0.58, *p* = 0.04) ^#^	(r = 0.62, *p* = 0.03) ^#^	*p* > 0.05
AIR TEMP (°C)	(r = −0.61, *p* = 0.03) *	(r= −0.72, *p* = 0.007) **	*p* > 0.05
PREC (mm)	*p* > 0.05	*p* > 0.05	*p* > 0.05

## Data Availability

The data that support the findings of this study are available from the corresponding author, [J.R.], upon reasonable request.
